# BIM_EL_ is a key effector molecule in oxidative stress-mediated apoptosis in acute myeloid leukemia cells when combined with arsenic trioxide and buthionine sulfoximine

**DOI:** 10.1186/1471-2407-14-27

**Published:** 2014-01-15

**Authors:** Yukie Tanaka, Takayuki Komatsu, Hiroko Shigemi, Takahiro Yamauchi, Yutaka Fujii

**Affiliations:** 1 Department of Molecular Biology and Chemistry, Faculty of Medical Sciences University of Fukui, 23-3 Shimoaizuki, Matsuoka, Eiheiji, Fukui, Japan; 2 Department of Microbiology and Immunology, School of Medicine, Aichi Medical University, 1-1 Yazako-Karimata, Nagakute, Aichi, Japan; 3 Department of Hematology and Oncology, Faculty of Medical Sciences, University of Fukui, 23-3 Shimoaizuki, Matsuoka, Eiheiji, Fukui, Japan

**Keywords:** Arsenic trioxide, Buthionine sulfoximine, Mitochondrial apoptosis, BIM_EL_, MCL1, BAX

## Abstract

**Abstracts:**

**Background**

Arsenic trioxide (ATO) is reported to be an effective therapeutic agent in acute promyelocytic leukemia (APL) through inducing apoptotic cell death. Buthionine sulfoximine (BSO), an oxidative stress pathway modulator, is suggested as a potential combination therapy for ATO-insensitive leukemia. However, the precise mechanism of BSO-mediated augmentation of ATO-induced apoptosis is not fully understood. In this study we compared the difference in cell death of HL60 leukemia cells treated with ATO/BSO and ATO alone, and investigated the detailed molecular mechanism of BSO-mediated augmentation of ATO-induced cell death.

**Methods**

HL60 APL cells were used for the study. The activation and expression of a series of signal molecules were analyzed with immunoprecipitation and immunoblotting. Apoptotic cell death was detected with caspases and poly (ADP-ribose) polymerase activation. Generation of intracellular reactive oxygen species (ROS) was determined using a redox-sensitive dye. Mitochondrial outer membrane permeabilization was observed with a confocal microscopy using NIR dye and cytochrome *c* release was determined with immunoblotting. Small interfering (si) RNA was used for inhibition of gene expression.

**Results**

HL60 cells became more susceptible to ATO in the presence of BSO. ATO/BSO-induced mitochondrial injury was accompanied by reduced mitochondrial outer membrane permeabilization, cytochrome *c* release and caspase activation. ATO/BSO-induced mitochondrial injury was inhibited by antioxidants. Addition of BSO induced phosphorylation of the pro-apoptotic BCL2 protein, BIM_EL_, and anti-apoptotic BCL2 protein, MCL1, in treated cells. Phosphorylated BIM_EL_ was dissociated from MCL1 and interacted with BAX, followed by conformational change of BAX. Furthermore, the knockdown of BIM_EL_ with small interfering RNA inhibited the augmentation of ATO-induced apoptosis by BSO.

**Conclusions**

The enhancing effect of BSO on ATO-induced cell death was characterized at the molecular level for clinical use. Addition of BSO induced mitochondrial injury-mediated apoptosis via the phosphorylation of BIM_EL_ and MCL1, resulting in their dissociation and increased the interaction between BIM_EL_ and BAX.

## Background

Arsenic trioxide (ATO) has been reported to be an effective therapeutic agent in both newly diagnosed and relapsed patients with acute promyelocytic leukemia (APL) [1,2]. This success has prompted an interest in understanding the molecular mechanisms of action underlying the clinical effectiveness of ATO. ATO is reported to induce apoptosis in leukemic promyelocytes [3,4]. ATO-induced apoptosis appears to be dependent on the intracellular redox homeostasis. In particular, the effectiveness of ATO in inducing to apoptosis is associated with an increased generation of intracellular reactive oxygen species (ROS) [5,6]. However, the antitumor effect of ATO is limited in other types of leukemia and solid tumor cells, since these cancer cell types have low susceptibility to ATO [7,8]. Previous studies suggest that the ineffectiveness of ATO in ATO-resistant tumors may be due to low ROS levels, preventing the triggering of effective apoptosis [5,9,10]. These early studies thus provide a rationale for utilizing ATO in combination with oxidative pathway modulators to extend the use of ATO for treating non-APL malignacies. Buthionine sulfoximine (BSO), which is known to effectively deplete cellular glutathione [11], is used to augment ATO-induced apoptosis [12-14]. However, the precise mechanism of BSO-mediated augmentation of ATO-induced apoptosis remains unclear. In particular, the molecular events in mitochondria involved in increased apoptotic susceptibility are unknown. In this study we investigated the detailed molecular mechanism of mitochondrial injury-mediated cell death by treating HL60 with ATO/BSO, compared with that with ATO alone. We report that the dissociation of BIM_EL_ and MCL1 and the subsequent interaction of BIM_EL_ and BAX play a critical role in BSO-mediated augmentation of ATO-induced apoptosis.

## Methods

### Reagents

ATO, BSO, n-acetylcysteine (NAC), dithiothreitol (DTT), SP600125, U0126, PD035901 and SB203580 were purchased from Sigma Chemical (St. Louis, MO, USA). The following antibodies were obtained from Cell Signaling Technology (Beverly, MA, USA): antibodies to the cleaved form of caspase 3 (C-cas3), caspase 9 (C-cas9), poly (ADP-ribose) polymerase (C-PARP); antibodies to normal and phosphorylated forms of MCL1 (MCL1, P-MCL1 at Ser159/Thr163), BCL2 (BCL2, P-BCL2 at Ser70), BIM (BIM, P-BIM at Ser69), JNK (JNK, P-JNK at Thr183/Tyr185), c-JUN (c-JUN, P-c-JUN at Ser63), p38 (p38, P-p38 at Thr180/Tyr182) and ERK1/2 (ERK1/2, P-ERK1/2 at Thr202/Tyr204); antibodies to actin, BAD, BID and BOK. Antibodies to BAK, ASK, and normal and phosphorylated forms of BCLxL (BCLxL, P-BCLxL at Ser62) were obtained from Abcam (Cambridge, MA, USA). Antibodies to mouse and human phosphorylated forms of ASK1 (P-ASK1 at Thr845 or at Thr838) [15] was provided by Dr. H. Ichijo, the University of Tokyo.

### Cell culture

The HL60 cell line, which was derived from peripheral blood cells of a 36-year old Caucasian female with APL, was obtained from ATCC (Manassas, VA, USA). Cells were maintained in RPMI-1640 medium supplemented with 10% heat inactivated fetal bovine serum.

### Cell viability

Cell viability was determined using a cell proliferation kit (XTT) (Roche Applied Sciences, Rotkreuz, Switzerland) as described elsewhere [16]. The half-maximal inhibitory concentration (IC_50_) was calculated using Graphpad PRISM software (GraphPad, San Diego, CA, USA). The nontoxic concentrations of various reagents were confirmed by the XTT test.

### Identification of apoptotic cell death

Apoptotic cells were identified using a cell death detection kit (Roche Applied Sciences) using mouse monoclonal antibodies against fragmented DNA and histones according to the manufacturer’s instructions.

### Determination of intracellular ROS level

The generation of intracellular ROS was determined using a redox-sensitive dye 5-(and-6)-chloromethyl-2′,7′-dichlorodihydrofluorescein diacetate probe (CM-H_2_DCFDA) (Molecular probes, Eugene, Oregon, USA) according to the manufacturer’s instructions.

### Determination of mitochondrial outer membrane permeability (MOMP)

Cells were incubated with NIR dye supplied in a NIR mitochondrial membrane potential assay kit (Abcam) and 1 μM Hoechst 33342 dye for 30 min at 37˚C. Stained cells were subjected to confocal microscopy (Leica TCS SP II, Wetzlar, Germany). The fluorescence ratio of the two dyes was determined for quantitative analysis of MOMP. The Leica confocal software, a MetaMorph ver.7.8 (Molecular Devices) was used for the analysis.

### Determination of cytochrome *c* release

The release of cytochrome *c* was determined using an ApoAlert cell fractionation kit (Clontech, Mountain View, USA). The cells were processed according to the manufacturer’s instructions and the concentration of released cytochrome *c* in the cytosolic fractions was determined by immunoblotting with anti-cytochrome *c* antibody.

### Immunoprecipitation and immunoblotting analysis

Cells were lysed in CHAPS buffer (10 mM Hepes, pH7.5, 150 mM NaCl, 2% CHAPS) or RIPA buffer (50 mM Tris–HCl, pH 8.0, 150 mM NaCl, 0.5% sodium deoxycholate, 0.1% sodium dodecyl sulfate, 1.0% NP-40) containing protease inhibitor cocktail (Nacalai Tesque, Kyoto, Japan) and phosphatase inhibitor cocktail I (Wako Pure Chemical, Osaka, Japan). Protein lysates (500 μg of protein) in CHAPS buffer were subjected to immunoprecipitation using an antibody to BAX 6A7 (BD Biosciences, San Jose, CA, USA), BAK Ab1 (Abcam), BIM (Cell Signaling Technology) or MCL1 (BD Pharmingen). The immunoprecipitates or whole cell lysates (10 μg of protein) were analyzed by sodium dodecyl sulfate-polyacrylamide gel electrophoresis on 7.5-15% gels and electroblotted onto nitrocellulose membranes (BIO-RAD, Hercules, CA, USA). Immunoblots were treated with primary and secondary antibodies and then analyzed using an ECL-Advance Western blotting kit (GE Healthcare, Little Chalfont, England). In several experiments, band intensities were quantified using a LAS4000 imaging system (GE Healthcare).

### Transfection with small interfering (si) RNA

HL60 cells (1 × 10^6^ cells) were transfected with two distinct siRNA (BIM#1 and BIM#2) specifically designed against human BIM_EL_, or with a control non-silencing siRNA (Silencer Select; Life Technologies, Carlsbad, CA, USA) using hemagglutinating virus supplied in a Japan-envelope vector kit (GenomONE-Si; Ishihara Sangyo, Osaka, Japan), according to the manufacturer’s instructions. The cells were used for ATO/BSO treatment 48 h after transfection with siRNA.

### Statistical analysis

Experimental values are represented as the mean ± standard deviation in triplicate. The experiments were carried out at least 3 times. The significance of difference between experimental and control groups was determined by the Student’s *t*-test. A value of *p* < 0.05 was considered statistically significant.

## Results

### BSO augments ATO-induced cell death via intracellular ROS generation

**Figure 1:**
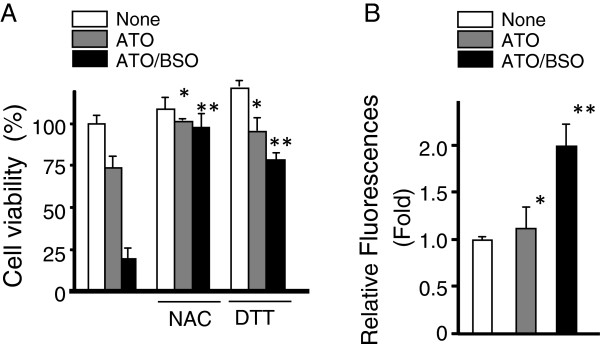
**BSO augments ATO-induced cell death via intracellular ROS generation. A**, HL60 cells were treated with ATO (3 μM) or ATO/BSO (3 μM/50 μM) in the presence or absence of NAC (5 mM) or DTT (0.6 mM) for 12 h. Cell viability was determined using a XTT assay. *p < 0.05, Treatment with ATO in the presence of NAC or DTT treatment vs. treatment with ATO in the absence of NAC and DTT; **p < 0.01, Treatment with ATO/BSO in the presence of NAC or DTT treatment vs. treatment with ATO/BSO in the absence of NAC and DTT. **B**, Cells were treated with ATO or ATO/BSO for 6 h. The levels of intracellular ROS were monitored. *p < 0.05, Treatment with ATO vs. treatment with none; **p < 0.01, Treatment with ATO/BSO vs. treatment with none.

The effect of BSO on ATO-induced cell death using HL60 cells was examined by determining cell viability (Figure 1A). BSO significantly augmented ATO-induced cell death. Approximately 80% of HL60 cells died when exposed to ATO in the presence of BSO, whereas ATO alone killed approximately 30% of the cells (p < 0.01, ATO alone or ATO/BSO vs. none). Since ATO-induced cell death is associated with generation of intracellular ROS [5,6], the effect of the antioxidants, NAC and DTT, on ATO/BSO-induced cell death was examined (Figure 1A). Antioxidants prevented ATO/BSO-induced cell death, suggesting that ROS play an important role in BSO-mediated augmentation of ATO-induced cell death. To confirm the ROS generation directly in ATO/BSO-treated cells, intracellular ROS generation was determined using a fluorescent probe, CM-H_2_DCFDA dye (Figure 1B). ATO/BSO treatment markedly increased the ROS generation, although ATO alone treatment did it only slightly.

### BSO augments ATO-induced cell death via ROS-mediated mitochondrial injury

**Figure 2:**
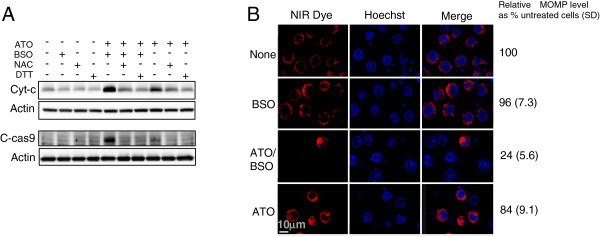
**BSO augments ATO-induced cell death via ROS-mediated mitochondrial injury. A**, After treatment as Figure 1A, the release of cytochrome *c* and the cleavage of caspase 9 were determined by immunoblotting. **B**, MOMP with NIR dye and Hoechst staining was analyzed by confocal microscopy. Magnification ×40. The results are presented as % untreated cells with SD. A typical result of 3 independent experiments is shown.

In the preceding section, BSO augmented ATO-induced cell death via intracellular ROS generation. To clarify involvement of ROS-mediated mitochondrial injury in BSO-mediated augmentation, the effect of BSO on the release of cytochrome *c*, a marker of mitochondrial injury, in ATO-treated cells was examined by immunoblotting. BSO significantly augmented ATO-induced cytochrome *c* release whereas ATO alone induced slight release of cytochrome *c* (Figure 2A). The cytochrome *c* release was abolished by antioxidants (Figure 2A). Further, BSO markedly augmented the activation of caspase 9, which is triggered by released cytochrome c and is involved in an early stage of mitochondrial apoptosis. On the other hand, the caspase 9 activation was hardly detected in ATO alone treatment. To confirm BSO-mediated mitochondrial injury, the effect of ATO/BSO treatment on MOMP was examined with a confocal microscope. Addition of BSO significantly reduced NIR stain in ATO-treated cells whereas ATO did it only minimally (Figure 2B). Thus, BSO was suggested to augment ATO-induced cell death via mitochondrial injury characterized by cytochrome c release, caspase 9 activation and MOMP reduction.

### BSO induces conformational change in BAX, but not in BAK

**Figure 3:**
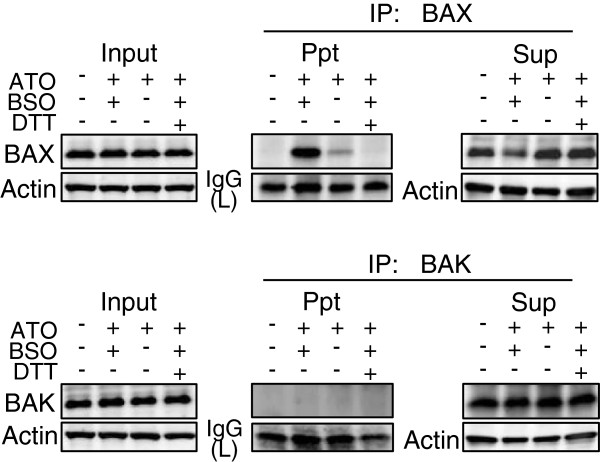
**BSO induces conformational change in BAX, but not BAK.** The conformational change was detected using immunoprecipitation and immunoblotting with an antibody to conformationally changed BAX or BAK. HL60 cells were treated with ATO/BSO or ATO for 12 h in the presence or absence of DTT. Actin and IgG light chain (IgG(L)) were used as the controls. A typical result of 3 independent experiments is shown. IP, immunoprecipitation; Ppt, immunoprecipitate; Sup, supernatant.

Since the two proapoptotic BCL2 effector proteins, BAX and BAK, play central roles in oxidative stress-mediated mitochondrial apoptosis [17,18], the effect of BSO addition on the conformational change of BAX and BAK in ATO-treated cells was examined by immunoprecipitation and immunoblotting. Immunoblotting analysis with anti-whole BAX antibody demonstrated no significant difference in total BAX expression between ATO/BSO and ATO alone treatment. However, an antibody which defines conformationally changed BAX immunoprecipitated much more BAX in ATO/BSO-treated cells than ATO alone-treated cells (Figure 3, upper panel). An anti-whole BAX antibody immunoprecipitated a lower level of BAX from the supernatant fraction of ATO/BSO-treated cells. Therefore, BSO was suggested to trigger conformational change of BAX in ATO/BSO treatment. In addition, the conformational change of BAX in ATO/BSO-treated cells was prevented by DTT as an antioxidant (Figure 3, upper panel).

Immunoprecipitation analysis using an antibody to whole BAK or conformationally changed BAK demonstrated no presence of conformationally changed BAK in either ATO/BSO or ATO alone treatment (Figure 3, lower panel).

### BSO induces phosphorylation of BIM_EL_ and MCL1 in mitochondria

**Figure 4:**
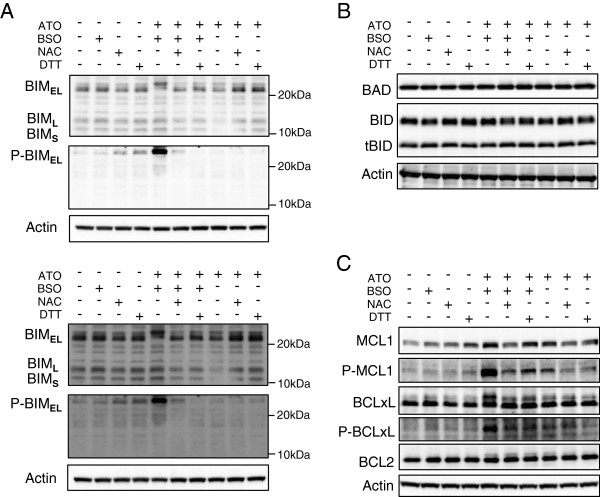
**BSO induces phosphorylation of BIM**_**EL **_**and MCL1 in mitochondria.** HL60 cells were treated with ATO/BSO or ATO in the presence or absence of NAC or DTT for 12 h. **A**. The expression and phosphorylation of BIM were determined by immunoblotting. The lower panel indicates a longer exposure of the same blot. **B, C**. The expression and phosphorylation of BAD, BID, tBID, MCL1, BCLxL and BCL2 were determined by immunoblotting.

A possibility was raised that the conformational change of BAX might be critical for BSO-mediated mitochondrial injury. Therefore, we examined the expression and activation of a series of BCL2-family proteins, which affect the conformational change of BAX [19,20]. First, the expression of BIM_EL,_ a proapoptotic protein in the BCL2 family, was analyzed by immunoblotting. Normal HL60 cells expressed a readily detectable level of the two major BIM isoforms, BIM_EL_ and BIM_L_ whereas they expressed a low level of the smallest isoform, BIM_S_ (Figure 4A upper panel). BIM_EL_ (23 kDa) underwent an electrophoretic mobility shift (24–26 kDa) following ATO/BSO treatment whereas the smaller isoform, BIM_L_ did not. BSO addition caused a high level of S^69^-phosphorylated BIM_EL_ (Figure 4A). The enhanced BIM_EL_ phosphorylation was abolished by NAC or DTT as antioxidants (Figure 4A). In contrast, neither mobility shift nor phosphorylation of BIM_EL_ was induced by ATO alone. There was no significant difference in the expression of the other pro-apoptotic proteins of the BCL2 family, BAD and BID between ATO/BSO and ATO treatment (Figure 4B).

Second, the effect of BSO addition on the expression and activation of MCL1, an anti-apoptotic protein of the BCL2 family, was examined. BSO addition augmented the expression and phosphorylation of MCL1 at Ser^159^ and/or Thr^163^, whereas ATO alone did it only minimally (Figure 4C). The BSO-mediated augmentation of MCL1 expression and phosphorylation was abolished by antioxidants. Similar augmentation was seen in phosphorylation of BCL_X_L (Figure 4C). In addition, there was no significant difference in the BCL2 expression in ATO/BSO treatment in the presence or absence of antioxidants (Figure 4C).

### BSO induces the dissociation of phosphorylated BIM_EL_ from MCL1

**Figure 5:**
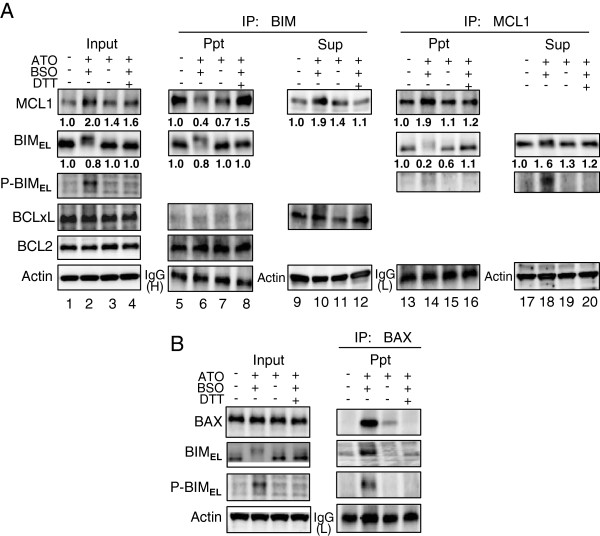
**BSO induces the dissociation of phosphorylated BIM**_**EL **_**from MCL1 and the interaction with BAX. A** and **B**, The dissociation of phosphorylated BIM_EL_ and MCL1, and the interaction with BAX were determined by immunoprecipitation and immunoblotting with antibodies to their normal and phosphorylated forms. Values were normalized to actin or IgG(L), respectively and represent relative changes compared with control. A typical result of 3 independent experiments is shown. IP, immunoprecipitation; Ppt, immunoprecipitate; Sup, supernatant.

Since MCL1 is a preferred binding partner for BIM [21], and BIM phosphorylation is known to influence the binding to prosurvival BCL2-family proteins, especially MCL1 [22,23], the phosphorylation of BIM_EL_ and/or MCL1 disrupting the complex formation between BIM_EL_ and MCL1 was examined using immunoprecipitation and immunoblotting. The MCL1-BIM_EL_ complex was detected in untreated control cells (Figure 5A, MCL1 lane 5). BSO augmented the phosphorylation of BIM_EL_ (Figure 5A, P-BIM_EL_ lane 2) and the expression of MCL1 (Figure 5A, MCL1 lane 2), but reduced the level of MCL1 that co-precipitated with BIM_EL_ (Figure 5A, MCL1 lane 6). The reduced interaction between BIM_EL_ and MCL1 was also confirmed using an MCL1-specific antibody (Figure 5A, BIM_EL_ lane 14). Furthermore, the interaction between MCL1 and phosphorylated BIM_EL_ was low (Figure 5A, P-BIM_EL_ lane 14). In contrast, ATO alone did not induce the phosphorylation of BIM_EL_ (Figure 5A, P-BIM_EL_ lane 3) but rather slightly reduced the amount of MCL1 that co-precipitated with BIM_EL_ (Figure 5A, MCL1 lane 7).

### BSO induces the interaction of phosphorylated BIM_EL_ with BAX

Since BIM promotes apoptosis through binding directly to BAX and inducing conformational changes [24,25], the interaction between BIM_EL_ dissociated from MCL1 and BAX in ATO/BSO treatment was examined using immunoprecipitation. As shown in Figure 5A, BSO reduced the amount of non-phosphorylated (basal) BIM_EL_ and increased the amount of BIM_EL_ slower migrating forms (phosphorylated BIM_EL_) in cell lysate (Figure 5B, left panel). The BIM_EL_ slower migrating form was detected in immunoprecipitates of BAX in ATO/BSO-treated cells but few in ATO alone-treated cells (Figure 5B, right panel). To confirm the interaction between BAX and phosphorylated BIM_EL_, BAX immunoprecipitates were analyzed by immunoblotting with an anti-phosphorylated BIM_EL_ antibody (Figure 5B, right panel). Phosphorylated BIM_EL_ was detected in BAX immunoprecipitates but not in ATO-treated cells. BSO was suggested to augment the interaction between phosphorylated BIM_EL_ and BAX.

### Silencing of BIM_EL_ with si RNA abolishes ATO/BSO-induced cell death

**Figure 6:**
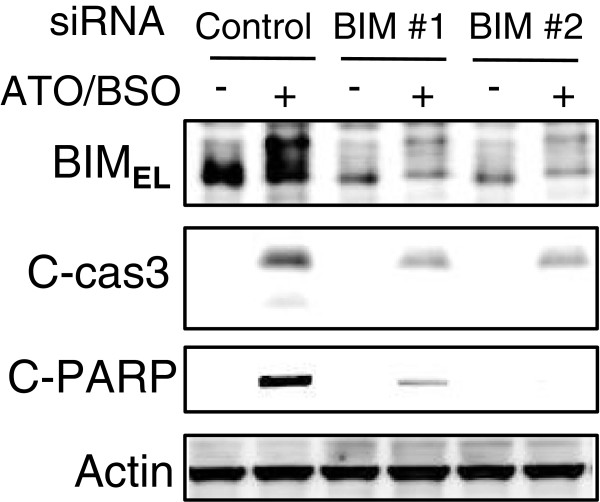
**Silencing of BIM**_**EL **_**with si RNA abolishes ATO/BSO-induced cell death.** The expression of BIM_EL_ and cleavage of caspase 3 and PARP were determined by immunoblotting. HL60 cells were transfected with two siRNAs designed against BIM_EL_ (BIM#1 and BIM#2) or control siRNA, incubated for 48 h, and treated with ATO/BSO for 12 h. A typical result of 3 independent experiments is shown. IP, immunoprecipitation; Ppt, immunoprecipitate.

To confirm the importance of BIM_EL_ in BSO-mediated augmentation of ATO-induced cell death, the effect of gene silencing of BIM_EL_ on ATO/BSO-induced cell death was examined (Figure 6). Transfection of HL60 cells with BIM_EL_-specific siRNA significantly decreased the expression level of BIM_EL_ whereas the negative control siRNA had no effect. BIM_EL_-specific siRNA but not control siRNA inhibited the cleavage of caspase 3 and PARP, markers of apoptosis, in ATO/BSO-treated cells, suggesting the critical role of BIM_EL_ in ATO/BSO-induced apoptosis.

### BSO triggers phosphorylation of MCL1 and BIM_EL_ via activation of JNK

**Figure 7:**
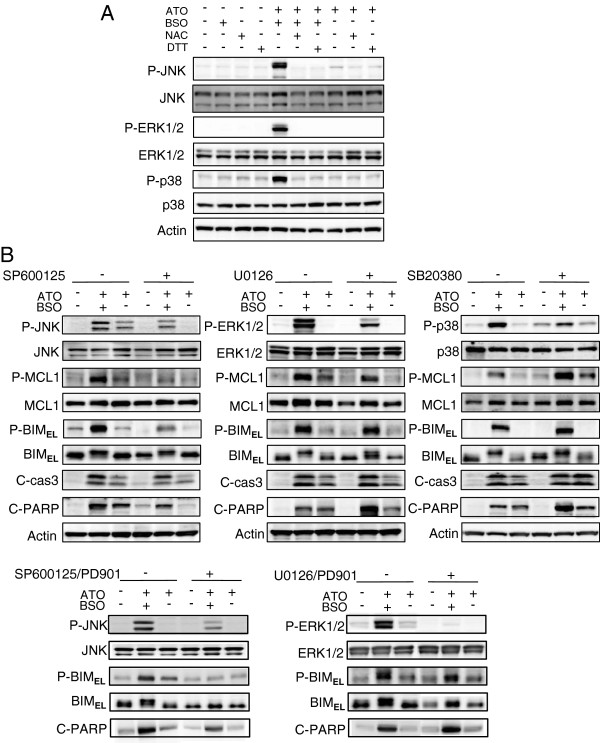
**BSO triggers phosphorylation of MCL1and BIM**_**EL **_**via activation of JNK. A**, The phosphorylation of JNK, ERK1/2 and p38 was determined by immunoblotting. HL60 cells were treated with ATO/BSO or ATO in the presence or absence of NAC or DTT for 12 h. **B**, The phosphorylation of BIM_EL_ and MCL1, and the cleavage of caspase 3 and PARP, were determined by immunoblotting. HL60 cells were treated with ATO/BSO or ATO in the presence of SP600125 (10 μM) as a JNK inhibitor, U0126 (2 μM) as an ERK1/2 inhibitor, or SB203580 (10 μM) as a p38 inhibitor, PD035901 (100 nM)as a MEK1/2 inhibitor for 12 h. A typical result of 3 independent experiments is shown.

To determine which mitogen-activated protein kinases (MAPKs) trigger phosphorylation of BIM_EL_ and MCL1 in response to ATO/BSO, the effect of BSO addition on ATO-induced activation of JNK, ERK1/2 and p38 was examined. As shown in Figure 7A, BSO augmented phosphorylation of JNK, ERK1/2 and p38 in ATO-treated cells. The phosphorylation of these proteins was largely abolished by the presence of antioxidants. Furthermore, the effect of a series of pharmacological inhibitors against MAPKs on BSO-induced phosphorylation of BIM_EL_ and MCL1 was examined (Figure 7B). SP600125, a JNK inhibitor, inhibited phosphorylation of MCL1 and BIM_EL_ (Figure 7B, left panel) whereas a p38 inhibitor (SB203580) augmented phosphorylation of BIM_EL_ and MCL1 compared to the untreated control (Figure 7B, right panel). An ERK1/2 inhibitor (U0126) did not affect the phosphorylation of BIM_EL_ and MCL1 (Figure 7B, middle panel). The phosphorylation of BIM_EL_ and MCL1 corresponded to the activation of caspase 3 and PARP (Figure 7B, upper panel). Further, the effect of a MEK1/2 inhibitor, PD035901, in combination with SP600125 or U0126 was examined (Figure 7B, lower panel). A combination of PD035901 and SP600125 completely blocked BSO-induced phosphorylation of BIM_EL_ present as slower migrating forms. A combination of PD035901 and U0126 did not affect BIM_EL_ S^69^ phosphorylation but blocked slower migrating forms. The phosphorylation of BIM_EL_ corresponded to the activation of PARP.

### BSO triggers activation of ASK1 and JNK and induces phosphorylation of BIM_EL_ and MCL1

**Figure 8:**
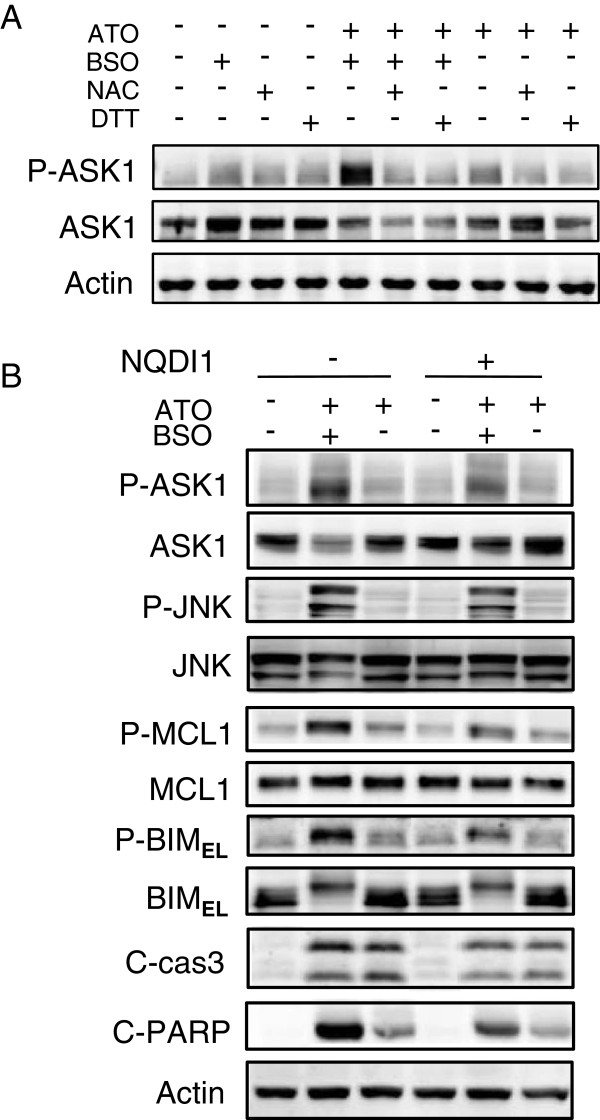
**BSO triggers activation of ASK1 and JNK and induces phosphorylation of BIM**_**EL **_**and MCL1. A**, The phosphorylation of ASK1 was determined by immunoblotting. HL60 cells were treated with ATO/BSO or ATO in the presence or absence of NAC or DTT for 12 h. **B**, The phosphorylation of JNK, BIM_EL_, and MCL1, and cleavage of caspase 3 and PARP, were determined by immunoblotting. HL60 cells were treated with ATO/BSO or ATO in the presence or absence of NQDI1 (10 μM) for 12 h. A typical result of 3 independent experiments is shown.

ASK1 is activated by ATO through ROS accumulation [26] and induces activation of JNK and p38 [27]. To confirm the involvement of ASK1 in BSO-mediated augmentation of ATO-induced cell death, the effect of BSO addition on the activation of ASK1 in ATO-treated cells was examined. Thr^838^ of ASK1 was markedly phosphorylated by BSO addition whereas no obvious phosphorylation was observed upon ATO alone (Figure 8A). The phosphorylation was inhibited by antioxidants (Figure 8A). Furthermore, the effect of an ASK1 inhibitor, NQDI1 [28], on the phosphorylation of JNK, MCL1 and BIM_EL_ was examined (Figure 8B). NQDI1 inhibited BSO-mediated phosphorylation of JNK, MCL1 and BIM_EL_ and the cleavage of caspase 3 and PARP (Figure 8B). BSO was suggested to activate ASK1 and induce the activation of MCL1, BIM_EL_, caspase 3 and PARP.

## Discussion

In the present study, we have demonstrated that BSO augments ATO-induced cell death in HL60 cells and that the augmentation is responsible for ROS-mediated mitochondrial apoptosis. The detailed molecular mechanism of BSO-mediated mitochondrial injury was studied by comparing ATO cell death in the presence or absence of BSO. We here report that BSO augments intracellular ROS production in mitochondria and induces a series of molecular events, such as conformational change of BAX, phosphorylation and dissociation of BIM_EL_ and MCL1, and interaction of BIM_EL_ and BAX.

Previously several groups showed that BSO decreased the levels of glutathione and enhanced ATO-induced apoptosis [29,30]. Chen et al. reported that ATO/BSO induced apoptosis in ATO-sensitive and insensitive leukemia cells through activation of JNK, which up-regulated death receptor (DR) 5 and the caspase 8 pathway [30]. However, they did not report the accumulation of ROS in ATO/BSO-induced apoptosis, nor the associated molecular events occurring in mitochondria. We have demonstrated that ATO/BSO induces the dissociation of BIM_EL_ from MCL1, and that its interaction with BAX plays a critical role in ATO/BSO-induced apoptosis via conformational changes in BAX. Our results demonstrate that BSO causes ROS-mediated mitochondrial injury, accompanied by cytochrome *c* release and reduced MOMP. This is the first report showing the involvement of ROS-mediated mitochondrial injury in BSO-mediated augmentation of ATO-induced apoptosis. Moreover, we show the pivotal role played by the pro-apoptotic molecule, BIM_EL_, in ATO/BSO-induced apoptosis, and confirm it by the finding that knockdown of BIM_EL_ abolishes ATO/BSO-induced apoptosis. The remarkable behavior of pro-apoptotic BIM_EL_ in mitochondria provides new insights into the molecular mechanism of ATO/BSO-induced apoptosis. Pro-apototic effects are reported to be associated with BIM_L_ activation [31]. However, we could not confirm the activation of BIM_L_ in this study. Rather, the role of BIM_EL_ might be more important than that of BIM_L_, although we do not exclude the involvement of BIM_L_ in ATO/BSO-induced apoptosis.

**Figure 9:**
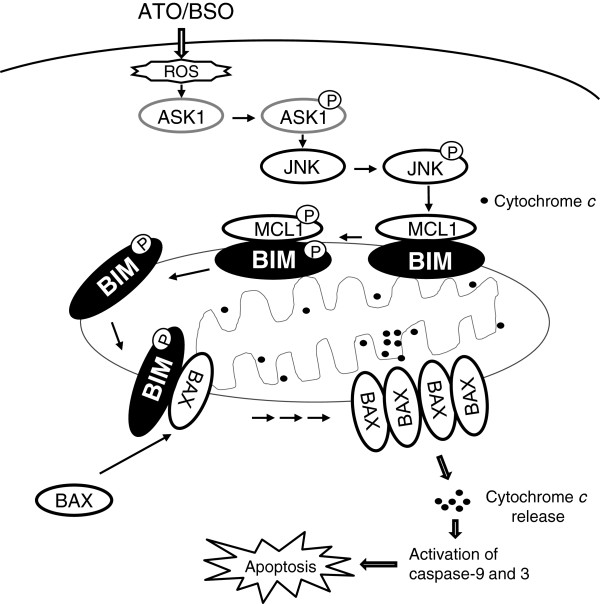
The putative molecular events occurring in mitochondria during ATO/BSO-induced apoptosis.

BSO induces phosphorylation of BIM_EL_ and MCL1 in ATO-treated cells. Phosphorylated BIM_EL_ is dissociated from MCL1 and interacts with BAX. The complex formation between phosphorylated BIM_EL_ and BAX triggers a conformational change in BAX, leading to the dysfunction of MOMP and the release of cytochrome *c*. Finally, ATO/BSO-treated cells undergo apoptosis via activation of cytochrome *c-*mediated caspase 9, caspase 3 and PARP. The putative molecular events occurring in mitochondria for BSO-mediated augmentation of ATO-induced apoptosis are summarized in Figure 9. There are several reports on the phosphorylation and dissociation of BIM and MCL1 [23,32,33]. The withdrawal of serum survival factors is reported to induce the phosphorylation of BIM at Ser^65^ (Ser^69^ in human) and dissociation from MCL1 and BCLxL [32]. In normal B cells treated with anti-IgM antibody, the phosphorylation of BIM at Ser^69^ has been reported to play a regulatory role in the release of MCL1 [33]. However, these studies reported that the dissociation of phosphorylated BIM from MCL1 is related to survival response, whereas our results demonstrate that the dissociation of BIM_EL_ from MCL1 leads to cell death in ATO/BSO-treated cells. The crucial role of BIM_EL_ phosphorylation in apoptosis has been reported previously for cell death induced by trophic factor deprivation [34] and diallyl trisulfide [35], although the dissociation of BIM_EL_ and MCL1 was not observed. There remained a possibility that phosphatase inhibition might be involved in the phosphorylation of a series of signal molecules.

The critical role of ASK1 in apoptosis induction has been reported [36-38]. We have found that BSO triggers activation of ASK1 in ATO-treated cells. The involvement of ASK1 activation in ATO/BSO-induced apoptosis is confirmed by using pharmacological inhibitors, although it must be confirmed by more specific technique. Yan et al. [26] reported that ASK1 is activated by ATO through ROS accumulation, and that it negatively regulates apoptosis in leukemia cells without activating JNK and p38. In contrast, our results clearly show that ASK1 activated by BSO causes the activation of JNK and p38. The difference between the two studies might be due to excessive ROS generation in response to ATO/BSO. ASK1 is a member of the MAPK kinase kinase family and activates JNK and p38 MAPKs in response to an array of stresses such as oxidative stress, endoplasmic reticulum stress and calcium influx [27]. It is reasonable that BSO activates ASK1 via oxidative stress and then activates JNK and p38. Inhibition of p38 with a pharmacological inhibitor induces the activation of caspase 3 and PARP in ATO/BSO-induced apoptosis, suggesting negative feedback of p38 against ATO/BSO-induced apoptosis. The precise role of ASK1 and MAPKs in ATO/BSO-mediated apoptosis must await further characterization.

## Conclusions

ATO/BSO combined treatment induces ROS-mediated mitochondrial apoptosis in HL60 cells. ATO/BSO-induced mitochondrial apoptosis is caused by successive BIM_EL_ alterations consisting of phosphorylation, dissociation from MCL1, and interaction with BAX. The enhancing effect of BSO on ATO-induced apoptosis was characterized at the molecular level for clinical use.

## Abbreviations

ATO: Arsenic trioxide; BSO: Buthionine sulfoximine; ATO/BSO: A combined treatment of ATO and BSO; ROS: Reactive oxygen species; APL: Acute promyelocytic leukemia; AML: Acute myeloid leukemia; BIMEL: BCL2-interacting mediator of cell death-extra long protein; MCL1: Myeloid cell leukemia-1 protein; BAX: BCL2-assocated X protein; BAK: BCL2-antagonist/killer protein; BAD: BCL2-associated death promoter protein; BID: BH3-interacting domain death agonist; tBID: Truncated BID; BCL2: B cell lymphoma 2 protein; BCLxL: BCL2-like X protein; JNK: c-JUN N-terminal kinase; ERK1/2: Extracellular signal-regulated kinase 1/2; ASK1: Apoptosis signal-regulating kinase 1.

## Competing interests

All of authors have no conflicts of interest to disclose.

## Authors’ contributions

Participated in research design: K, T. Conducted experiments: T, S, K. Contributed new reagents or analytic tools: T, S. Performed data analysis: K, T, Y. Wrote or contributed to the writing of the manuscript: K, T, Y, S, F. All authors read and approved the final manuscript.

## Pre-publication history

The pre-publication history for this paper can be accessed here:

http://www.biomedcentral.com/1471-2407/14/27/prepub
